# Modeling of Electrical Heating and Cooling for Carbon Textile Reinforced Concrete

**DOI:** 10.3390/ma18051078

**Published:** 2025-02-27

**Authors:** Annette Dahlhoff, Michael Raupach

**Affiliations:** Institute of Building Materials Research (IBAC), RWTH Aachen University, Schinkelstr. 3, 52062 Aachen, Germany

**Keywords:** carbon-textile-reinforced concrete, electrical heating, thermal behavior, temperature, modeling

## Abstract

Carbon-textile-reinforced concrete (CTRC) is increasingly being used in the construction industry as a high-performance composite material combining non-metallic textile reinforcement with concrete. Known for its exceptional characteristics such as tensile strength, density, and durability, CTRC also exhibits electrical conductivity, enabling efficient electrical heat generation within building components. This study develops and validates a thermal model to predict the temperature evolution of electrically heated CTRC, incorporating Newton’s law of cooling and Joule’s heating principle. The proposed model segments the temperature development into three distinct phases: heating, constant, and cooling. The temperature calculation accounts for these phases, their boundary conditions, and material-specific parameters, which were determined through laboratory experiments. For the investigated CTRC material combinations, the model accurately predicts temperature profiles, demonstrating strong agreement between experimental and calculated results. Moreover, significant variations in electrical power requirements were observed among the tested materials. The investigated impregnation materials of the carbon textile reinforcement (CTR) significantly influence contact quality and resulting temperature behavior. This research bridges material science and thermal performance, expanding the potential for CTRC use in electrically heated construction solutions.

## 1. Introduction

**Figure 1:**
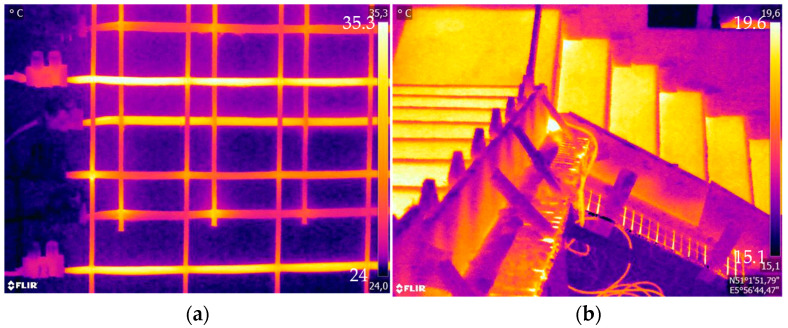
Example of electrically heated CTRC: (**a**) Acrylate-impregnated CTR; (**b**) CTRC stairs.

Carbon-textile-reinforced concrete (CTRC) is an innovative construction material composed of high-performance carbon-textile reinforcement (CTR) embedded in a concrete matrix. This innovative combination facilitates the construction of thin, durable, and resource-efficient structures with remarkable material properties, including tensile strengths of up to 4200 N/mm^2^ [1,2,3]. In addition to their mechanical advantages, carbon fibers also are electrically conductive [4]. By utilizing the electrical and high thermal conductivity of carbon fibers along their fiber direction, the development of electrically heated CTRC can be facilitated. Exemplarily, heated CTRC is depicted in Figure 1. This multifunctional technology operates as a heating element in building components by employing Joule’s heating principle [4,5,6]. When an electrical voltage is applied to the conductor, heat is generated, leading to an increase in temperature.

Research in building materials has explored various applications of electrical heating technology within construction, investigating its effective integration into different systems [7,8,9]. Electrical heating systems have been investigated in various concrete applications, particularly for pavement heating [7]. As early as the 1960s, electrical cables or pipes containing heated fluids were embedded in bridge decks and ramps to prevent icing [7]. In 1981, a bridge deck heating system was implemented using gravity-operated heat pipes in conjunction with a geothermal heat exchanger, further advancing the research field [7]. Subsequent research focused on enhancing the efficiency of electrical heating systems by leveraging electrically conductive concrete. In this context, electrical conduction within concrete matrix is facilitated by ionic movement [7]. To further improve conductivity, metallic or other conductive fibers, as well as particles, can be added to the concrete matrix [7,10]. Chung et al. investigated the self-heating efficiency of various conductive additives, evaluating their performance starting form an initial temperature of 19 °C [11]. Their findings demonstrated that uncoated fibers and steel fibers were the most effective materials for achieving self-heating, apart from flexible graphite [11].

The application of electrically conductive concrete has been further investigated in various studies, including its implementation in the Roca Spur Bridge in 2001 by Tuan [7,12]. In this case, the system demonstrated effective de-icing capabilities, achieving snow removal for depths up to 257 mm with a power density of up to 431 W/m^2^ [7,12,13]. Yehia et al. [14,15,16] conducted additional research by varying the ratio of steel fibers in conductive concrete. Through small-scale heating experiments, Yehia et al. demonstrated that an average power density of 48 W/m^2^ was sufficient to raise the slab temperature from −1.1 °C to 15.6 °C within 30 min [14,15,16]. Building upon this, Zhao et al. [17] investigated the use of carbon fiber wires as heating elements in concrete, employing both finite element modeling and laboratory experiments. Their results indicated that a power density of 1134 W/m^2^ could increase the concrete temperature from −25 °C to 0 °C within 2.5 h [17].

Karalis et al. [6] conducted experimental and numerical investigations on mineral-impregnated carbon-fiber-reinforced grids for the efficient and lower-power de-icing of geopolymer concrete surfaces. Their findings revealed that a surface temperature of 45 °C could be achieved by applying a voltage of 2.2 V, requiring a heating power of only 95.6 W/m^2^ over a 30 min period [6].

Further investigations have focused on the use of electrically heated concrete to accelerate curing, to enable in situ curing of concrete structures in cold environments, and to assess the impact of various additives [18,19]. He et al. [18] investigated the influence of incorporation of nano-carbon black into ordinary concrete, while Salim et al. [19] examined various dosages of multi-walled carbon nanotubes and carbon fibers, ranging from 0 to 1% by weight of cement. Additionally, Gomis et al. [20] studied conductive cement pastes with carbonaceous material through experimental and modeling approaches, demonstrating a correlation between laboratory results and mathematical predictions. Moreover, Rao et al. [21] experimentally analyzed material properties, such as density and thermal conductivity, of electrically heated concrete containing steel fibers and graphite.

Furthermore, CTRC can serve as an electrical resistance heating element, enabling functionalities such as radiant heating or thermal energy storage in concrete components, while also providing condensation protection [22,23]. This potential was demonstrated by employing electrically heated CTRC for radiant heating in wall systems and stairs [24,25]. Furthermore, the heat generated by electrically heated CTRC has been shown to enhance the hydration process of concrete and can be applied for de-icing purposes in outdoor facilities like parking areas, airport runways, and bridges [9,26]. To analyze electrically heated textile-reinforced concrete, Xu et al. [27] performed both finite element analysis and experimental investigations on the electrothermal properties of carbon/glass fiber hybrid textile-reinforced concrete, validating its efficiency for de-icing applications via heat generation. Xu et al. [27] assumes uniform temperature distribution along carbon rovings due to their length exceeding the spacing, periodic symmetry of the rovings in the cross-section, and constant physical properties of all materials and neglects the impact of contact thermal resistance for their numerical simulation.

**Figure 2:**
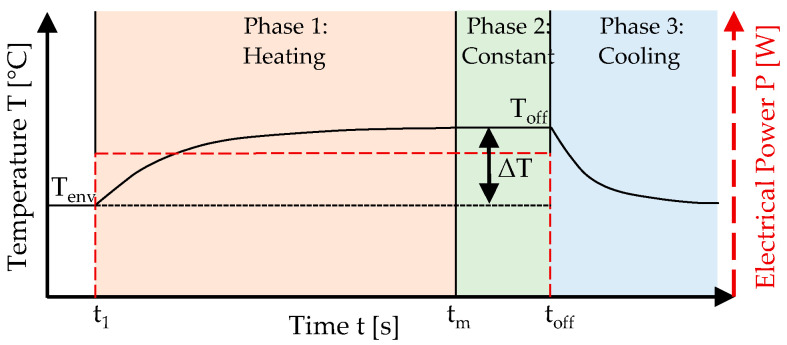
Schematic overview of heat generation in electrically heated CTRCs.

Accurate prediction of thermal development in electrically heated CTRC, based on applied electrical power and material-specific parameters, is crucial for its application and design. To achieve this, a thermal model was developed in this study, drawing upon fundamental physical principles based on Newton’s law of cooling [28,29]. Newton’s law of cooling was therefore inverted and adapted to describe the heating process. The model allows for the prediction of temperature changes using specific material parameters, thus eliminating the need for further laboratory testing. In the initial phase, the thermal model was derived, and the material parameters necessary for predicting temperature behavior were identified. Based on this, experimental laboratory tests were conducted to determine the material-specific parameters for the material combinations tested. These combinations were then compared based on the identified parameters. In addition, the experimentally measured temperature change was compared with the values predicted by the proposed thermal model. Figure 2 presents the schematic heat generation of electrically heated CTRC, demonstrating the temperature development and electrical power as a function of time.

The temperature development can be divided into three distinct phases: Phase 1 (heating), Phase 2 (constant temperature), and Phase 3 (cooling). In Phase 1, at time t_1,_ a constant electrical power is applied, initiating the heating process of the CTRC. From time t_m_ onwards, the maximum temperature, relative to the applied voltage, is reached and remains constant as long as the voltage is continuously applied. Once the voltage is switched off at time t_off_, the cooling phase begins, marking the transition to Phase 3. The analysis assumes a constant connection quality; however, if this condition is not maintained over extended periods, a reduction in temperature is expected during Phase 2. To assess the thermal behavior of electrically heated CTRC, this paper develops a thermal model that predicts temperature changes based on applied power and material parameters. By combining simulations with experimental validation, it offers a comprehensive approach to understanding CTRC’s performance, distinguishing it from existing studies and minimizing the need for additional testing.

## 2. Materials

**Figure 3:**
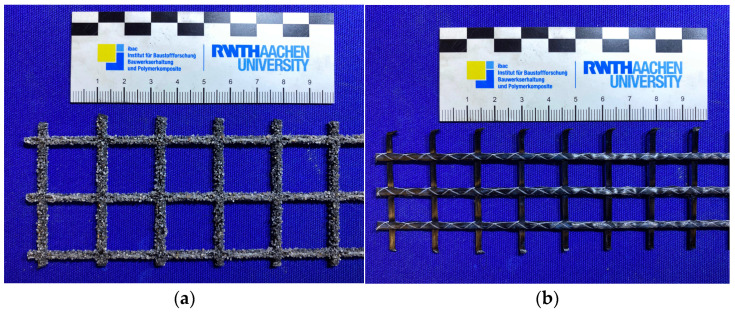
Investigated carbon-textile reinforcements: (**a**) CTR-EP-Sand; (**b**) CTR-P.

In this study, four combinations of carbon-textile-reinforced composites were examined, comprising two commercially available CTRs and two commercially available repair mortars, according to [30,31]. For the investigation, each of the two CTRs was combined with the two different repair mortars. The material properties of the CTRs are modified by the variations in the impregnation materials and geometrical configurations. Among the investigated CTRs, CTR-EP-Sand is impregnated with epoxy resin and further surface-modified through an additional coating of epoxy resin and quartz sand. CTR-EP-Sand is characterized by a roving axis distance of 21 mm and a uniform roving cross-section of 0.91 mm^2^ in both the warp and weft directions. Additionally, a CTR impregnated with polystyrene, designated as CTR-P, was analyzed. This textile contains a roving axis distance of 12/16 mm and a roving cross-section of 1.81 mm^2^ in both directions. The CTRs examined in this study are shown in Figure 3, with their material properties summarized in Table 1.

Two polymer-modified, cement-based wet spray mortars designed for the repair of concrete structures with a maximum grain size of 2 mm were analyzed in this study. The repair mortars are classified according to [30,31], and their differences are shown, based on the material properties, in Table 2.

## 3. Thermal Model

Based on Newton’s law of cooling, the thermal model was further developed. To ensure the model’s applicability and computational feasibility, several simplifications and assumptions were made during its formulation. One important assumption was the neglect of contact thermal resistance between the carbon-textile reinforcement (CTR) and the mortar. Additionally, the model does not account for potential changes in material properties over time due to the influence of temperature. This simplification assumes that the thermal properties of the materials remain constant during the temperature variations considered in the model. While this assumption is generally valid for short-term temperature fluctuations, it may not capture long-term effects or more extreme temperature ranges, where material degradation or changes in properties could become significant. For this, the mathematical equations governing each phase, as shown in Figure 2, are developed and described in the following sections.

### 3.1. Phase 3: Cooling

For the cooling phase, Newton’s law of cooling was applied. The system under consideration consists of a temperature-changing body made of mortar and exposed to ambient air. If the supplied electrical power is turned off, the cooling process of the electrically heated CTRC begins. Consequently, thermal energy is released to the surroundings at a rate that can be calculated using Newton’s law of cooling, as described by the differential equation referred to as Equation (1). During the cooling process, no additional energy is supplied. As a result, the concrete acts as the sole cooling body. The defined abbreviations refer to all of the following equations and times according to Figure 2.

(1) $$m_{c}c_{p\_c}\frac{dT}{dt}=-h_{c}A_{c}(T-T_{env})$$

with:

$m_{c}$ = mass of the sample for cooling [kg];
$c_{p\_c}$ = specific heat capacity for cooling [J/kg °C];
T = surface temperature [°C];
t = time [s];
$h_{c}$ = energy transfer coefficient towards the outside for cooling [W/m^2^ °C];
$A_{c}$ = surface of the sample [m^2^];
$T_{env}$ = environmental temperature [°C].

To model the cooling process T_c_(t), Equation (1) is integrated using the limit condition t = t_off_, where T = T_off_. This condition represents the point at which the electrical power P is switched off. The integration results in Equation (2):

(2) $$T_{c}\left(t\right)=T_{env}+\left(T_{off}-T_{env}\right)\cdot e^{-\frac{h_{c}A_{c}}{m_{c}c_{p\_c}}\cdot \left({t-t}_{off}\right)}$$

### 3.2. Phase 1: Heating

For the heating phase, Newton’s law of cooling was applied and extended. The analyzed system involves a body made of CTR and mortar subjected to temperature variations and exposed to ambient air. Consequently, the parameters are defined for the entire body. The model incorporates the electrical power input into Newton’s law of cooling. At the beginning, the CTRC is heated from an initial specimen temperature T_env_ at time t_1_ to a temperature T at time t due to the Joules heating effect. This temperature increase is determined by the applied electrical power P, minus the rate of energy loss due to temperature differences. This relationship can be calculated using Newton’s law of cooling, cf. Equation (3) [20,28,29]. The defined abbreviations refer to all of the following equations and times according to Figure 2.

(3) $$m_{h}c_{p\_h}\frac{dT}{dt}=P-h_{h}A_{h}(T-T_{env})$$

with:

$m_{h}$ = mass of the sample [kg];
$c_{p\_h}$ = specific heat capacity for heating [J/kg °C];
T = sample temperature [°C];
t = time [s];
P = applied electrical power [W];
$h_{h}$ = energy transfer coefficient towards the outside for heating [W/m^2^ °C];
$A_{h}$ = surface of the sample [m^2^];
$T_{env}$ = environmental temperature [°C].

To apply this relationship for calculating the temperature increase T_h_(t) in electrically heated CTRC, the equation is integrated using the initial condition t = t_1_ for T = T_env_, which represents the time at which the potential difference is first applied, cf. Equation (4) [20]:

(4) $$T_{h}\left(t\right)=T_{env}+\frac{P}{h_{h}A_{h}}\left[1-e^{-\frac{h_{h}A_{h}}{m_{h}c_{p\_h}}\cdot \left({t-t}_{1}\right)}\right]$$

### 3.3. Phase 2: Constant Temperature

Phase 2 begins when the temperature remains constant and no further temperature increase is observed. A steady-state temperature is assumed under constant electrical power, continuing indefinitely t → ∞. Under this condition, Equation (4) can be adapted to Equation (5):

(5) $$T=T_{env}+\frac{P}{h_{h}A_{h}}$$

This indicates that at steady-state temperature, the supplied electrical power is equal to the energy loss, as calculated using Newton’s law of cooling. Therefore, Equation (5) can be transformed to evaluate the electrical power P(T), cf. Equation (6):

(6) $$P(T)=h_{h}A_{h}(T-T_{env})$$

### 3.4. Determination of Material-Specific Parameters

In order to apply the equations developed in Section 3.1, Section 3.2 and Section 3.3, it is necessary to determine the material-specific parameters h, A, m, and c_p_.

In the first step, the parameters h and A for each material combination are determined based on the constant temperature Phase 2. By applying Equation (6), the linear correlation is shown in Equation (7):

(7) $$P=h_{h}A_{h}\cdot ∆T$$

**Figure 4:**
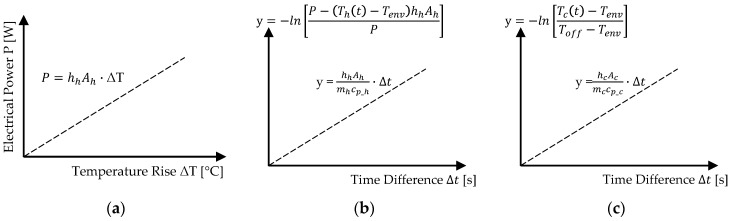
Linear correlation for determining the material-specific parameters: (**a**) h_h_A_h_; (**b**) heating: h_h_A_h_/m_h_c_p_h_; (**c**) cooling: h_c_A_c_/m_c_c_p_c_.

This relationship allows for the determination of h_h_A_h_ as the slope of a linear equation, cf. Figure 4a. The equation is experimentally evaluated for different material combinations by analyzing the temperature difference ΔT relative to the applied electrical power P, providing the required material-specific parameters.

The determined values of h_h_A_h_ are then used to transform the temperature increase T_h_(t), as described in Equation (4), into a linear equation dependent on m and c_p_h_, which need to be determined. Equation (8) presents this linearized form, where the slope of the correlation for Phase 1 is given by h_h_A_h_/m_h_c_p_h_, depicted in Figure 4b:

(8) $$-ln\left[\frac{P-\left(T_{h}\left(t\right)-T_{env}\right)h_{h}A_{h}}{P}\right]=\frac{h_{h}A_{h}}{m_{h}c_{p\_h}}\cdot \left({t-t}_{1}\right)$$

For Phase 3 (cooling), the material parameters are determined by transforming the cooling equation, cf. Equation (2), into a linear form. The resulting equation, shown in Equation (9) and illustrated in Figure 4c, defines a linear correlation with an intercept at the origin:

(9) $$-ln\left[\frac{T_{c}\left(t\right)-T_{env}}{T_{off}-T_{env}}\right]=\frac{h_{c}A_{c}}{m_{c}c_{p\_c}}\cdot \left({t-t}_{off}\right)$$

These transformations allow for the experimental determination of the material-specific parameters. The resulting linear correlations, summarized in Figure 4, arise from the mathematical reformulation of the underlying exponential relationships. By applying logarithmic transformations to Newton’s law of cooling and the heating equation, the originally nonlinear behavior is converted into a linear form, enabling a straightforward determination of material parameters.

## 4. Experimental Methods

### 4.1. Methods

To determine the specific material parameters and validate the developed thermal model, experimental laboratory tests were conducted on the material combinations outlined in Section 2. Four material combinations were examined, based on two CTRs and two repair mortars. For this purpose, thermal tests were conducted on rectangular CTRC specimens, in which electrical connections were installed and electrical power was applied. In these experiments, the target temperature difference ΔT, defined as the difference between the heated specimen temperature T and the specimen temperature under environmental conditions T_env_, was incrementally increased from ΔT = 5 °C to ΔT = 40 °C. For each temperature level, a separate specimen was evaluated. The temperature range was selected to cover the relevant application scenarios for the electrically heated CTRC, while ensuring that no experimental issues, such as degradation of the connections due to excessive heat, would occur. An overview of the experimental series is provided in Table 3.

### 4.2. Experimental Setup and Testing Procedure

**Figure 5:**
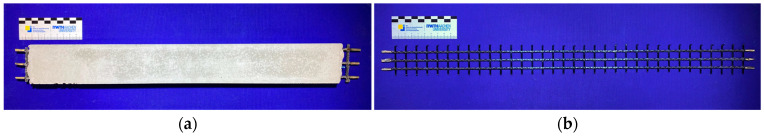
CTRC rectangular specimen: (**a**) example of CTRC-EP-M1 specimen; (**b**) position of thermal elements on the CTR, exemplarily in CTR-P.

To determine the thermal behavior of electrically heated CTRC, rectangular CTRC specimens with integrated thermal elements were manufactured, as shown in Figure 5. The specimens measured 500 mm × 60 mm × 20 mm (L × W × H), with a constant mortar cover on both sides and a single layer of CTR. For this, CTR grids of three fiber strands in the warp direction were cut from larger textile-reinforcement grids. The CTRC specimens were hand-laminated using a solid-to-water weight ratio of 1:0.13 for both mortar materials. Subsequently, the fresh mortar properties were evaluated in accordance with [36,37,38]. Additionally, standard prism sets were produced to determine the flexural and compressive strengths corresponding to the respective thermal test date in accordance with [39].

**Figure 6:**
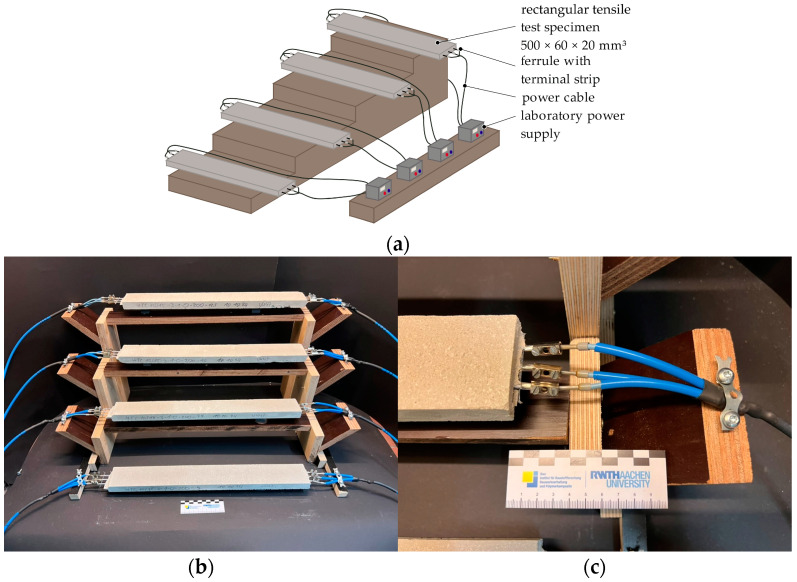
Test setup for the thermal tests on rectangular CTRC specimen: (**a**) schematic drawing; (**b**) overview of test setup; (**c**) example of the connection detail.

The specimens were demolded after one day, stored in water until the seventh day after production, and subsequently stored at a temperature of 20 ± 1 °C and a relative humidity of 55 ± 19% until testing at the age of 28 days after production. Prior to testing, the fiber strand ends were contacted using ferrules and terminal strips. For the CTR-EP-Sand specimens, the sand coating on the fiber strand ends was removed before establishing the electrical contact. On the day of testing, the specimens were connected to laboratory power supply, depicted in Figure 6. Four specimens were simultaneously tested within the test setup.

**Figure 7:**
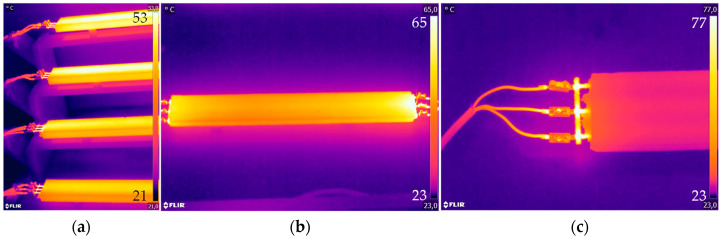
Example of heated CTRC: (**a**) thermal overview test setup; (**b**) CTRC-EP-M1 for ΔT = 27.5 °C; (**c**) connection detail—CTRC-EP-M1 for ΔT = 20 °C.

During testing, the target temperature levels were set by adjusting the voltage, based on temperature measurements obtained using a Flir E96 thermal imaging camera, cf. Figure 7. During the testing, the surface temperature of the specimen was recorded approximately every 5 min. The heating tests were stopped after 20 min of constant temperature in Phase 2, and the cooling phase ended when T_r_ was reached.

## 5. Results

### 5.1. Heating Tests

To evaluate the application of the proposed thermal model in direct comparison with the laboratory experiments, Table 4 and Table 5 summarize the required electrical power to achieve the respective temperature difference ΔT. The results demonstrate that the desired temperature levels could be achieved during the experiments. However, a comparison between the investigated CTRs reveals differences in the required electrical power for the same temperature levels. For example, to achieve a temperature rise of ΔT = 35 °C, the required electrical power for CTRC-EP-M1 was P = 58.3 W, whereas for CTRC-P-M1, it was significantly lower, at P = 34 W. For increasing temperature rise ΔT, the discrepancy between the electrical power between EP and P for identical mortars become more pronounced. As shown in Table 4 and Table 5, ΔP_CTRC-EP-M1-CTRC-P-M1_ starts at 1.0 W and progressively rises to a maximum of 38.9 W at the highest temperature stage. In contrast, no significant differences were observed between the investigated mortars M1 and M2.

**Figure 8:**
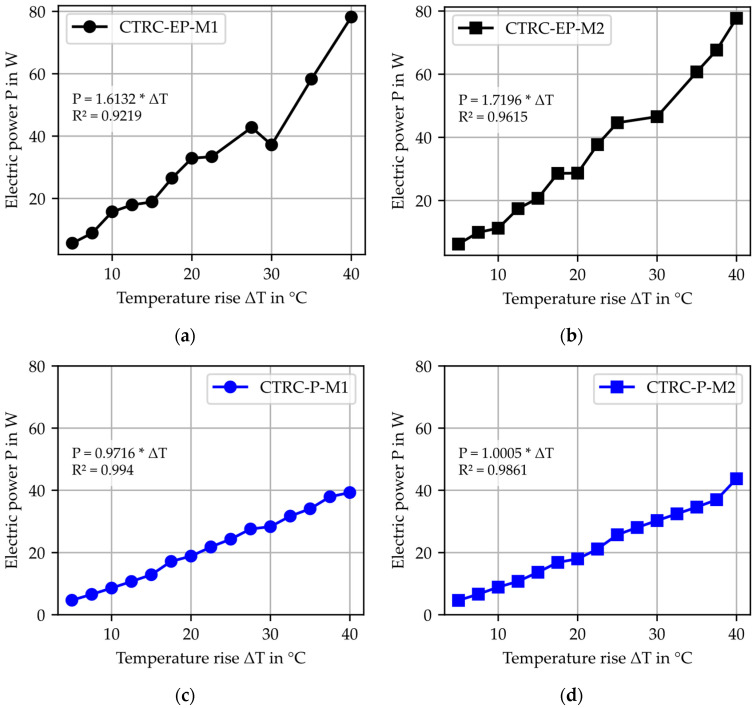
Linear correlation of the experimental data according to Equation (7): (**a**) CTRC-EP-M1; (**b**) CTRC-EP-M2; (**c**) CTRC-P-M1; (**d**) CTRC-P-M2.

To visualize the temperature development as a function of electrical power and to determine h_h_A_h_ as the slope for the material combinations based on Equation (7), the linear relationships of the investigated material combinations are depicted in Figure 8. A high coefficient of determination R^2^ validates the mathematical foundation described in Equation (7) and confirms the accuracy of the measurements without power consumption. The results of h_h_A_h_ further highlighted the described differences between the CTRs. In Figure 8a,b, the coefficient of determination R^2^ of the linear function was lower for CTRC-EP compared to CTRC-P. Additionally, significant outliers were observed in the electric power used. The authors hypothesize that these differences in electrical resistance and the reduced coefficient of determination are related to the variations in the contact area of CTR. The epoxy resin coating, combined with additional sanding for CTR-EP-Sand, weakens the contact and the epoxy resin acts as an insulating layer. Outliers resulting from insufficient contact have been excluded from further analysis, cf. Table 4. Outliers are identified as experimental series in which the deviation between the electrical power derived from the applied voltage and current and the value calculated from the linear regression exceeds 16%.

Furthermore, the experimental series revealed that CTR-P specimens heat up significantly faster than CTRC-EP. In addition to the impregnation materials of CTR, the authors assume that the observed differences in temperature increase may be attributed to the material-specific reinforcement geometries of the CTR, such as fiber cross-section, as shown in Table 1. For comparison, the textile cross-section of CTR-P is three times as large as that of CTR-EP-Sand.

**Figure 9:**
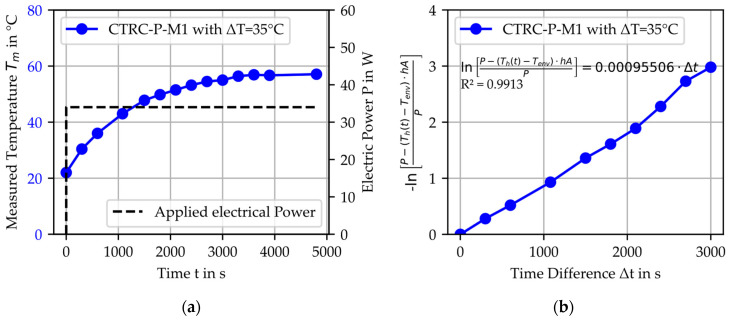
Example of heated CTRC-P-M1 with ΔT = 35 °C: (**a**) measured temperature with the applied electrical power; (**b**) linear correlation for determining the material-specific parameter h_h_A_h_/m_h_c_p_h_.

To apply the developed thermal model and to calculate the temperature profile using Equation (4) in comparison to the measured temperature, cf. Figure 9a, the material-specific parameter h_h_A_h_/m_h_c_p_h_ was determined for the experimental series based on Equation (8). This is exemplified in Figure 9b, and the results of the fitting, along with the coefficient of determination, are summarized in Table 6 and Table 7.

**Figure 10:**
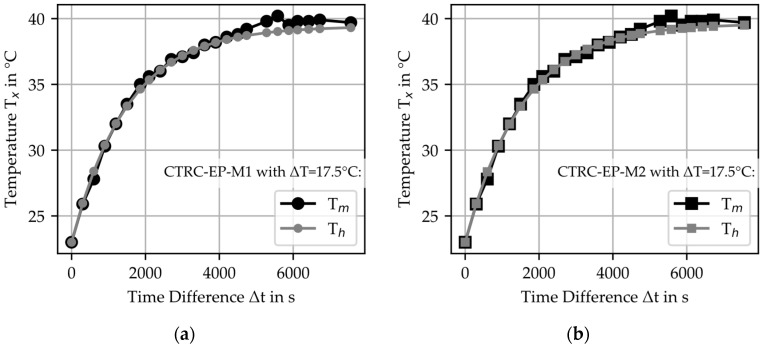

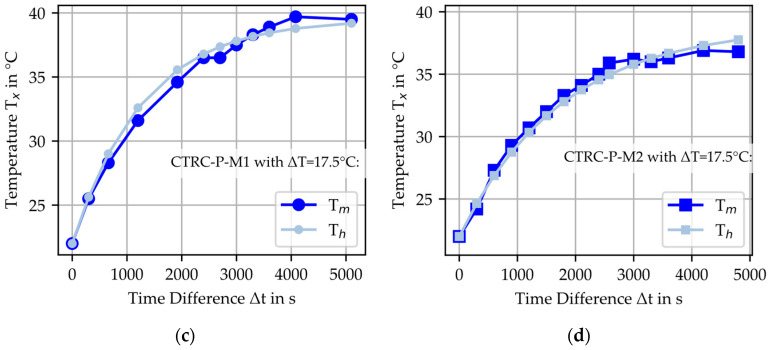
Comparison of calculated temperature T_h_ with the measured temperature T_m_ for heated CTRC specimen with ΔT = 17.5 °C: (**a**) CTRC-EP-M1; (**b**) CTRC-EP-M2; (**c**) CTRC-P-M1; (**d**) CTRC-P-M2.

Using the determined parameters, the measured temperatures were compared with the calculated temperature profiles for each test. As an example, the temperature development for the material combinations with a temperature difference of ΔT = 17.5 °C is shown in Figure 10. The results indicate a high level of agreement between the calculated T_h_ and measured T_m_ values, which confirms the applicability of the thermal model.

To quantitatively analyze the comparison between calculated and measured temperatures, the coefficient of determination was calculated for the experimental series. The coefficient was determined based on the ratio between the measured temperature T_m_ and the calculated temperature T_h_ at each measurement time. The results, as shown in Table 8 and Figure 10, demonstrate a high level of agreement, with mean R^2^ exceeding 0.94.

Additionally, the analysis reveals a trend that the agreement is greater for larger temperature differences ΔT than for smaller ΔT. This can be attributed to the electrical power being set too high, which resulted in an overshoot of the temperature increase. This overshoot is also evident in the temperature profiles. In the initial phase, the temperature rise gradient of the measured temperature is increased to the calculated temperature. The authors assume that this effect is more pronounced at lower temperature ΔT.

The modeling, based on the experimental results of the material parameters, involved two consecutive regression steps, each evaluated using the coefficient of determination R^2^. By multiplying the R^2^ values from both regression steps, the overall model accuracy R^2^_mean_ was evaluated. The mean R^2^ value across all investigated material combinations was determined to be 0.92. Based on this, the authors define 0.92 as the threshold for sufficiently precise model predictions. An ${R^{2}}_{T_{h}-T_{m}}$ value above this limit indicates a high agreement between calculated and measured temperature values, suggesting that deviations remain within acceptable limits for engineering applications. For ${R^{2}}_{T_{h}-T_{m}}$ values between 0.90 and 0.92, minor discrepancies may arise due to experimental uncertainties or idealized assumptions in the model, yet the predictions remain reliable. If ${R^{2}}_{T_{h}-T_{m}}$ falls below 0.90, further investigation into the experimental setup and modeling assumptions may be necessary to improve accuracy.

The four material combinations investigated in this study serve as an initial approach for exploring the behavior of the thermal model. However, to fully evaluate the model’s limitations and its applicability under varying conditions, further testing with additional material combinations and under extreme environmental conditions is required. Nevertheless, the results presented in this study provide a solid basis for the practical implementation of the thermal model in construction, contributing valuable insights for future research and advancements in the field.

### 5.2. Cooling Tests

**Figure 11:**
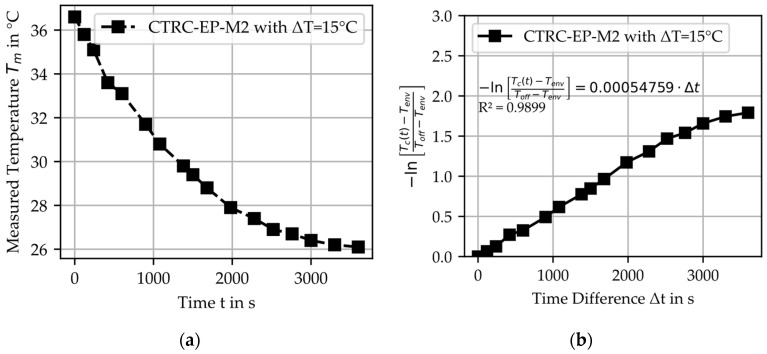
Example of heated CTRC-EP-M2 with ΔT = 15 °C: (**a**) measured temperature T_m_; (**b**) linear correlation for determining the material-specific parameter h_c_A_c_/m_c_c_p_c_.

To analyze the cooling behavior by comparing measured and calculated temperature profiles, Figure 11a illustrates the measured cooling curve exemplarily for CTRC-EP-M2. The cooling behavior reveals no significant differences between the investigated material combinations. Based on the thermal model for cooling, the material-specific parameter h_c_A_c_/m_c_c_p_c_ was determined for the material combinations, as shown in Figure 11b and summarized in Table 9. The fitting results demonstrate a remarkable agreement for the investigated materials, as evidenced by the coefficient of determination R^2^.

**Figure 12:**
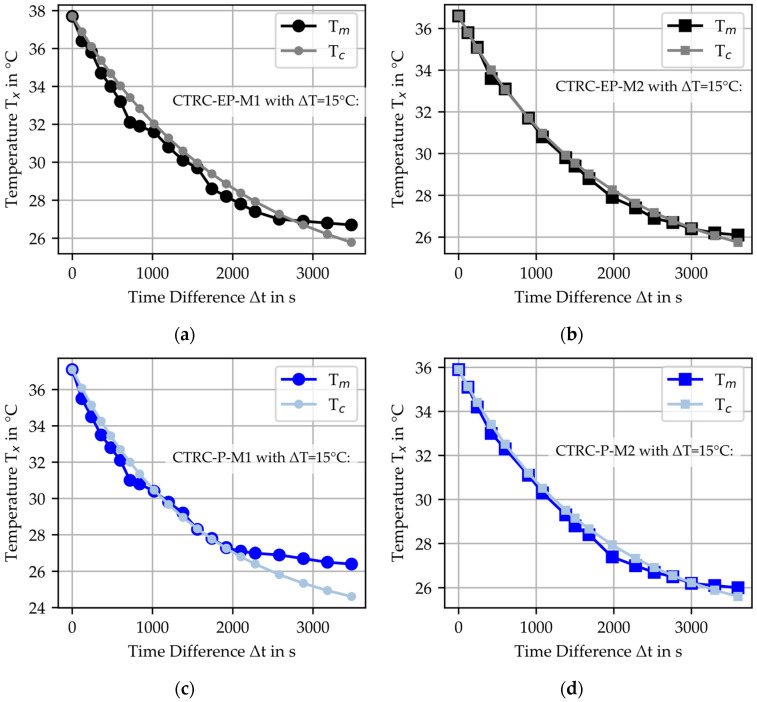
Comparison of calculated temperature T_c_ with the measured temperature T_m_ for cooling CTRC specimen with ΔT = 15 °C: (**a**) CTRC-EP-M1; (**b**) CTRC-EP-M2; (**c**) CTRC-P-M1; (**d**) CTRC-P-M2.

Using the determined parameters, the cooling curve for T_c_ was calculated based on Equation (2), cf. Figure 12. Additionally, the coefficient of determination R^2^ between the calculated T_c_ and the measured T_m_ was evaluated and summarized in Table 10. The results demonstrate a high level of agreement between the calculated and measured values, confirming the applicability of the thermal model. For ${R^{2}}_{T_{h}-T_{m}}$ values greater than 0.94, the deviations between the measured and calculated temperature values can be considered sufficiently accurate for practical applications. This threshold is based on the multiplication of the R^2^ values from the linear regressions of the experimental data for the material parameter, resulting in a minimum combined R^2^_mean_ value of approximately ~0.94.

When comparing the materials, the most significant differences between T_m_ and T_c_ were observed for CTRC-P-M1 at lower temperatures (~26 °C). The authors hypothesize that this can be attributed to the decreased R^2^ values obtained during the calculation of h_c_A_c_/m_c_c_p_c_ and that the measured values may need to decline further.

## 6. Conclusions and Outlook

Within this framework, a thermal model based on Newtons’ law of cooling was developed and validated for predicting the temperature of electrically heated CTRCs. Laboratory experiments demonstrated the model’s capability to accurately replicate measured temperature profiles during both heating and cooling phases. The methodology was evaluated using four material combinations, comprising two mortars and two CTRs, one impregnated with epoxy resin and the other with polystyrene. The thermal behavior of these materials was compared with respect to their heating and cooling processes. Furthermore, material-specific parameters essential for the application of the thermal model were determined for each combination, highlighting the suitability and potential of the proposed approach.

The key findings of this study can be summarized as follows:

- Overall, a thermal model was developed based on Newtons’s law of cooling with Joule’s heating principle. The temperature development is segmented into three distinct phases: Phase 1 (heating phase), Phase 2 (constant phase), and Phase 3 (cooling phase). The model formulation for temperature calculation accounts for these phases along with their respective boundary conditions and geometries. In addition, the prediction of the temperature profile for electrically heated CTRCs requires material-specific parameters. These parameters can be determined through laboratory experiments for each material, derived as fitting results.
- The thermal model successfully predicts the temperature development in CTRC, achieving a high correlation with experimental data. The comparison between calculated and measured temperature profiles demonstrates an average coefficient of determination of R^2^ = 0.94 for heating and R^2^ = 0.98 for cooling, confirming the applicability.
- For the investigated CTRC material combinations, the material-specific parameters h_h_A_h_ and h_i_A_i_/m_i_cp__i_ for heating and cooling were determined. For the heating phase, Newton’s law considers the body consisting of both the CTR and the mortar, while for the cooling phase, only the mortar cools as the body interacting with the ambient temperature. Using the material-specific parameters, the temperature profiles were calculated.
- The results indicate remarkable variations in the electrical power required to attain equivalent temperature levels depending on the CTR material. For instance, for a temperature level of ΔT = 35 °C, CTRC-EP-M1 required P = 58.3 W, whereas CTRC-P-M1 only requires P = 34 W. This effect becomes more pronounced at higher temperature levels, with differences in electrical power between the reinforcements reaching up to ΔP = 38.9 W. The results suggest that the impregnation of the CTR significantly impacts contact quality, which, in turn, affects both the electrical resistance and the temperature development within the CTRC.
- In contrast to the CTR type, the variation in mortar type (M1 vs. M2) had no significant effect on the electrical power required for heating. Both mortars exhibited comparable thermal behavior, as seen in the heating tests.

These findings are crucial, as they demonstrate the potential of the thermal model to accurately predict the temperature distribution in electrically heated CTRCs within building components. By determining the material-specific parameters of the examined CTRCs, this research enhances the understanding of their behavior and expands the possibilities for integrating this innovative method into construction applications. Further research should focus on developing a comprehensive database of material combinations and geometries, enabling the application of the thermal model for designing electrically heated CTRCs with a variety of materials. Additionally, it is important to investigate potential limitations of the model in extreme environmental conditions. Furthermore, laboratory experiments could investigate the impact of additional impregnation materials on contact quality and electrical resistance. Moreover, the thermal model could be tested and validated under varying temperature differences during the cooling process. For the practical implementation of electrically heated CTRC applications, future research should investigate their long-term stability and durability, particularly under repeated heating and cooling cycles, to assess their performance over extended durations. These proposed investigations could be integrated into the research project MultiTexBridge (grant number KK5038622KT4), funded by Federal Ministry for Economic Affairs and Climate Action (BMWK—Bundesministerium für Wirtschaft und Klimaschutz).

## Figures and Tables

**Table 1:** Material parameters of the carbon-textile reinforcement according to the manufacturer’s specification and measurements [32,33].

Reinforcement	Roving	Roving	Textile	Titer	Average Tensile Strength ± Standard Deviation ^(1)^ (Warp Direction)
	Axis Distance	Cross-Section	Cross-Section		
	Longitudinal/Transversal				
[-]	[mm]	[mm^2^]	[mm^2^/m]	[tex]	[MPa]
CTR-EP-Sand	21/21	0.91/0.91	43/43	1600/1600	4200 ± 215
CTR-P	12/16	1.81/0.45	142/25	3220/800	2920 ± 95

^(1)^ Measurements with 5 specimens performed at the Institute of Building Materials Research (ibac), RWTH Aachen University.

**Table 2:** Material parameters as mean values with standard deviation of the repair mortars according to manufacturer’s specification and measurements [34,35].

Mortar	Compressive Strength ^(1)^	Bending Strength ^(1)^	Young’s Modulus
[-]	[MPa]	[MPa]	[GPa]
M1	56.3 ± 1.5	9.6 ± 0.6	21
M2	62.6 ± 1.4	8.3 ± 0.4	27

^(1)^ Measurements with 3 specimens performed at the Institute of Building Materials Research (ibac), RWTH Aachen University after 28 days of curing, based on the manufacturing process.

**Table 3:** Overview of the investigated experimental test series.

Test Series	CTR	Mortar	Heating	Cooling
			Temperature Difference ΔT Related to T_env_ in °C	
CTRC-EP-M1	CTR-EP-Sand	M1	ΔT = 5 °C to 40 °C in 2.5 °C steps	15
CTRC-EP-M2	CTR-EP-Sand	M2	ΔT = 5 °C to 40 °C in 2.5 °C steps	15
CTRC-P-M1	CTR-P	M1	ΔT = 5 °C to 40 °C in 2.5 °C steps	15
CTRC-P-M2	CTR-P	M2	ΔT = 5 °C to 40 °C in 2.5 °C steps	15

**Table 4:** Summary of heating tests results for CTRC-EP: applied electrical voltage, current, and power.

Temperature Rise ΔT	CTRC-EP-M1			CTRC-EP-M2		
	Voltage	Current	Electric Power	Voltage	Current	Electric Power
[°C]	[V]	[A]	[W]	[V]	[A]	[W]
5	5.4	1.1	5.7	5.5	1.1	6.2
7.5	6.5	1.4	8.9	6.8	1.5	9.9
10	8.5	1.9	15.7	7.2	1.6	11.2
12.5	9.8	1.8	17.9	8.8	2.0	17.4
15	9.0	2.1	18.9	11.8	2.1	24.7
17.5	12.8	2.1	26.5	13.0	2.2	28.6
20	13.0	2.5	32.9	12.8	2.2	28.7
22.5	13.0	2.6	33.4	14.5	2.6	37.7
25 ^(1)^	17.0	3.5	59.8	16.0	2.8	44.6
27.5 ^(2)^	15.5	2.8	42.8	17.0	3.1	52.0
30	13.0	2.9	37.2	19.0	2.5	46.6
32.5 ^(1),(2)^	16.0	3.9	62.1	17.0	3.5	59.2
35	17.5	3.3	58.3	16.5	3.7	60.7
37.5 ^(1)^	21.0	4.0	84.0	19.0	3.6	67.6
40	20.0	3.9	78.2	18.5	4.2	77.7

^(1)^ Test specimen CTRC-EP-M1 was excluded from further analysis due to insufficient contact. ^(2)^ Test specimen CTRC-EP-M2 was excluded from further analysis due to insufficient contact.

**Table 5:** Summary of heating tests results for CTRC-P: applied electrical voltage, current, and power.

Temperature Rise ΔT	CTRC-P-M1			CTRC-P-M2		
	Voltage	Current	Electric Power	Voltage	Current	Electric Power
[°C]	[V]	[A]	[W]	[V]	[A]	[W]
5	2.8	1.7	4.7	2.8	1.7	4.6
7.5	3.3	2.0	6.6	3.3	2.0	6.6
10	3.8	2.3	8.6	3.9	2.3	8.9
12.5	4.2	2.6	10.7	4.2	2.6	10.8
15	4.6	2.8	12.9	4.7	3.0	14.1
17.5	5.3	3.2	17.2	5.3	3.2	16.9
20	5.6	3.4	18.9	5.5	3.3	18.0
22.5	6.0	3.6	21.8	5.9	3.6	21.1
25	6.5	3.9	25.4	6.5	4.0	25.7
27.5	6.7	4.1	27.5	6.8	4.1	28.0
30	6.7	4.2	28.3	7.0	4.3	30.2
32.5	7.2	4.4	31.7	7.3	4.4	32.4
35	7.4	4.6	34.0	7.6	4.6	34.6
37.5	7.8	4.9	37.9	7.7	4.8	37.0
40	7.9	5.0	39.3	8.9	4.9	43.7

**Table 6:** Results of fitting the experimental data from the heating tests using Equation (8) for CTRC-EP.

Temperature Rise ΔT	CTRC-EP-M1		CTRC-EP-M2	
	$\frac{h_{h}A_{h}}{m_{h}c_{p\_h}}$	${R^{2}}_{\frac{h_{h}A_{h}}{m_{h}c_{p\_h}}}$	$\frac{h_{h}A_{h}}{m_{h}c_{p\_h}}$	${R^{2}}_{\frac{h_{h}A_{h}}{m_{h}c_{p\_h}}}$
[°C]	[10^−3^/s]	[-]	[10^−3^/s]	[-]
5	1.02	0.9187	4.09	0.9496
7.5	6.23	0.9094	6.61	0.9209
10	3.21	0.8037	11.36	0.9148
12.5	7.97	0.9879	10.69	0.9853
15	1.04	0.9441	11.34	0.9190
17.5	6.64	0.9928	6.48	0.9951
20	4.98	0.9887	10.97	0.9696
22.5	10.67	0.9744	5.29	0.9427
25	-	-	4.30	0.9109
27.5	4.23	0.9885	-	-
30	10.49	0.9807	6.88	0.9803
32.5	-	-	-	-
35	17.11	0.8552	15.27	0.9569
37.5	-	-	11.79	0.9948
40	4.69	0.9758	48.05	0.9463

$\frac{h_{h}A_{h}}{m_{h}c_{p\_h}}$: linear coefficient according to Equation (8). ${R^{2}}_{\frac{h_{h}A_{h}}{m_{h}c_{p\_h}}}$: Coefficient of determination R^2^ for the linear coefficient$\;\frac{h_{h}A_{h}}{m_{h}c_{p\_h}}$.

**Table 7:** Results of fitting the experimental data from the heating tests using Equation (8) for CTRC-P.

Temperature Rise ΔT	CTRC-P-M1		CTRC-P-M2	
	$\frac{h_{h}A_{h}}{m_{h}c_{p\_h}}$	${R^{2}}_{\frac{h_{h}A_{h}}{m_{h}c_{p\_h}}}$	$\frac{h_{h}A_{h}}{m_{h}c_{p\_h}}$	${R^{2}}_{\frac{h_{h}A_{h}}{m_{h}c_{p\_h}}}$
[°C]	[10^−3^/s]	[-]	[10^−3^/s]	[-]
5	11.91	0.9924	6.24	0.9709
7.5	10.82	0.9858	9.02	0.9135
10	16.15	0.9189	5.39	0.9175
12.5	20.46	0.9178	8.50	0.9280
15	15.00	0.9434	8.45	0.9763
17.5	7.72	0.9391	5.69	0.9563
20	8.71	0.9703	13.27	0.9413
22.5	7.02	0.9718	14.43	0.8821
25	9.42	0.9508	8.74	0.9898
27.5	8.79	0.9759	10.54	0.9582
30	15.98	0.9419	12.06	0.9713
32.5	12.42	0.9581	11.31	0.9725
35	9.55	0.9884	7.11	0.9602
37.5	9.01	0.9969	9.45	0.9765
40	13.18	0.973	5.05	0.9179

$\frac{h_{h}A_{h}}{m_{h}c_{p\_h}}$: Linear coefficient according to Equation (8). ${R^{2}}_{\frac{h_{h}A_{h}}{m_{h}c_{p\_h}}}$: Coefficient of determination R^2^ for the linear coefficient$\;\frac{h_{h}A_{h}}{m_{h}c_{p\_h}}$.

**Table 8:** Comparison of the calculated temperature $T_{h}\left(t\right)$ from Equation (4) with the measured temperature T_m_ based on the coefficient of determination R^2^.

Temperature Rise ΔT	${R^{2}}_{T_{h}-T_{m}}$			
	CTRC-EP-M1	CTRC-EP-M2	CTRC-P-M1	CTRC-P-M2
[°C]	[-]			
5	0.9392	0.9403	0.9555	0.9782
7.5	0.9708	0.9323	0.9780	0.9639
10	0.9544	0.9080	0.9594	0.9809
12.5	0.9921	0.9515	0.9569	0.9669
15	0.9778	0.9364	0.9510	0.9794
17.5	0.9958	0.9963	0.9902	0.9891
20	0.9950	0.9543	0.9913	0.9743
22.5	0.9856	0.9876	0.9927	0.9497
25	-	0.9195	0.9862	0.9961
27.5	0.9945	-	0.9958	0.9817
30	0.9084	0.9819	0.9801	0.9903
32.5	-	-	0.9886	0.9904
35	0.9778	0.9804	0.9956	0.9945
37.5	-	0.9957	0.9988	0.9909
40	0.9661	0.7477	0.9891	0.9552

${R^{2}}_{T_{h}-T_{m}}$: Coefficient of determination R^2^ based on the calculated temperature $T_{h}\left(t\right)$, calculated with Equation (4), compared to the measured temperature T_m_.

**Table 9:** Results of fitting the experimental data from the cooling tests using Equation (9).

Cooling ΔT	CTRC-EP-M1		CTRC-EP-M2		CTRC-P-M1		CTRC-P-M2	
	$\frac{h_{c}A_{c}}{m_{c}c_{p\_c}}$	${R^{2}}_{\frac{h_{c}A_{c}}{m_{c}c_{p\_c}}}$	$\frac{h_{c}A_{c}}{m_{c}c_{p\_c}}$	${R^{2}}_{\frac{h_{c}A_{c}}{m_{c}c_{p\_c}}}$	$\frac{h_{c}A_{c}}{m_{c}c_{p\_c}}$	${R^{2}}_{\frac{h_{c}A_{c}}{m_{c}c_{p\_c}}}$	$\frac{h_{c}A_{c}}{m_{c}c_{p\_c}}$	${R^{2}}_{\frac{h_{c}A_{c}}{m_{c}c_{p\_c}}}$
[°C]	[10^−3^/s]	[-]	[10^−3^/s]	[-]	[10^−3^/s]	[-]	[10^−3^/s]	[-]
15	4.78	0.9431	5.47	0.9899	6.24	0.9789	5.58	0.9812

$\frac{h_{c}A_{c}}{m_{c}c_{p\_c}}$: linear coefficient according to Equation (9). ${R^{2}}_{\frac{h_{c}A_{c}}{m_{c}c_{p\_c}}}$: coefficient of determination R^2^ for the linear coefficient$\;\frac{h_{c}A_{c}}{m_{c}c_{p\_c}}$.

**Table 10:** Comparison of the calculated temperature $T_{c}\left(t\right)$from Equation (2) with the measured temperature T_m_ based on the coefficient of determination R^2^.

Cooling ΔT	${R^{2}}_{T_{h}-T_{m}}$			
	CTRC-EP-M1	CTRC-EP-M2	CTRC-P-M1	CTRC-P-M2
[°C]	[-]			
15	0.9831	0.9967	0.9822	0.9949

## Data Availability

The original contributions presented in this study are included in the article. Further inquiries can be directed to the corresponding author.

## Abbreviations

The following abbreviations are used in this manuscript:

CTRC	Carbon textile reinforced concrete
CTR	Carbon textile reinforcement
